# Weaker HLA Footprints on HIV in the Unique and Highly Genetically Admixed Host Population of Mexico

**DOI:** 10.1128/JVI.01128-17

**Published:** 2018-01-02

**Authors:** Maribel Soto-Nava, Santiago Avila-Ríos, Humberto Valenzuela-Ponce, Claudia García-Morales, Jonathan M. Carlson, Daniela Tapia-Trejo, Daniela Garrido-Rodriguez, Selma N. Alva-Hernández, Thalía A. García-Tellez, Akio Murakami-Ogasawara, the International HIV Adaptation Collaborative, Simon A. Mallal, Mina John, Mark A. Brockman, Chanson J. Brumme, Zabrina L. Brumme, Gustavo Reyes-Teran, the HIV MexNet Group

**Affiliations:** a Center for Research in Infectious Diseases, National Institute of Respiratory Diseases, Mexico City, Mexico; b Microsoft Research, Redmond, Washington, USA; c Vanderbilt University, Nashville, Tennessee, USA; d Murdoch University, Perth, Australia; e Faculty of Health Sciences, Simon Fraser University, Burnaby, BC, Canada; f British Columbia Centre for Excellence in HIV/AIDS, Vancouver, BC, Canada; Emory University

**Keywords:** adaptation, Canada/United States, HLA, immunogenetics, Mexico, human immunodeficiency virus

## Abstract

HIV circumvents HLA class I-restricted CD8^+^ T-cell responses through selection of escape mutations that leave characteristic mutational “footprints,” also known as HLA-associated polymorphisms (HAPs), on HIV sequences at the population level. While many HLA footprints are universal across HIV subtypes and human populations, others can be region specific as a result of the unique immunogenetic background of each host population. Using a published probabilistic phylogenetically informed model, we compared HAPs in HIV Gag and Pol (PR-RT) in 1,612 subtype B-infected, antiretroviral treatment-naive individuals from Mexico and 1,641 individuals from Canada/United States. A total of 252 HLA class I allele subtypes were represented, including 140 observed in both cohorts, 67 unique to Mexico, and 45 unique to Canada/United States. At the predefined statistical threshold of a q value of <0.2, 358 HAPs (201 in Gag, 157 in PR-RT) were identified in Mexico, while 905 (534 in Gag and 371 in PR-RT) were identified in Canada/United States. HAPs identified in Mexico included both canonical HLA-associated escape pathways and novel associations, in particular with HLA alleles enriched in Amerindian and mestizo populations. Remarkably, HLA footprints on HIV in Mexico were not only fewer but also, on average, significantly weaker than those in Canada/United States, although some exceptions were noted. Moreover, exploratory analyses suggested that the weaker HLA footprint on HIV in Mexico may be due, at least in part, to weaker and/or less reproducible HLA-mediated immune pressures on HIV in this population. The implications of these differences for natural and vaccine-induced anti-HIV immunity merit further investigation.

**IMPORTANCE** HLA footprints on HIV identify viral regions under intense and consistent pressure by HLA-restricted immune responses and the common mutational pathways that HIV uses to evade them. In particular, HLA footprints can identify novel immunogenic regions and/or epitopes targeted by understudied HLA alleles; moreover, comparative analyses across immunogenetically distinct populations can illuminate the extent to which HIV immunogenic regions and escape pathways are shared versus population-specific pathways, information which can in turn inform the design of universal or geographically tailored HIV vaccines. We compared HLA-associated footprints on HIV in two immunogenetically distinct North American populations, those of Mexico and Canada/United States. We identify both shared and population-specific pathways of HIV adaptation but also make the surprising observation that HLA footprints on HIV in Mexico overall are fewer and weaker than those in Canada/United States, raising the possibility that HLA-restricted antiviral immune responses in Mexico are weaker, and/or escape pathways somewhat less consistent, than those in other populations.

## INTRODUCTION

CD8^+^ cytotoxic T lymphocytes (CTLs) recognize short, HIV-derived peptide epitopes presented by human leukocyte antigen (HLA) class I molecules on the surface of infected cells, thereby modulating early viremia control (1, 2) and the establishment of the viral set point (3). HLA-restricted CTLs also exert strong evolutionary pressure on HIV *in vivo*, promoting viral adaptation through the selection of escape mutations (4) that interfere with epitope processing (5), prevent binding of the viral peptide to HLA (6, 7), or affect HLA-peptide recognition by the T-cell receptor (8, 9). Early observations that CTL escape in HIV tended to occur along predictable mutational pathways in persons responding to a given HLA-restricted viral epitope (10–13) led to the development of statistical approaches to systematically identify HLA-associated polymorphisms (HAPs), also known as HLA-associated “footprints,” on HIV, using large population-based data sets of viral sequences linked to HLA types (13). These analyses, which identify amino acids that are statistically overrepresented (or underrepresented) among persons expressing a given HLA allele while correcting for host and viral genetic confounders (14, 15), confirmed the broadly reproducible nature of CTL escape in HIV (16–20). These studies also revealed that certain HLA-associated footprints can be host population specific due to substantial immunogenetic variation across human populations. For example, even though HIV subtype B predominates in Japan, Canada, the United States, and Australia, two-thirds of HAPs in Japan are not observed in the latter epidemics (17) due to the unique HLA distribution of the Japanese population.

Identification of HLA-associated footprints is relevant to HIV vaccine design. An effective vaccine will need to elicit sustained immune responses capable of recognizing genetically diverse viral strains from which HIV cannot escape (ideal) or can only escape at substantial fitness cost (15). One promising strategy is to select immunogenic yet mutationally constrained viral regions as vaccine antigens (e.g., see references 21 and 22), which can be further optimized for natural sequence coverage (e.g., using mosaic designs [23, 24]). HLA footprints are vaccine relevant because they identify HIV regions under significant and consistent immune pressure by particular HLA-restricted CTLs (i.e., immunogenic regions) and the common mutational pathways that HIV uses to evade them. As such, evaluation of HLA footprints in concert with information on sequence conservation, mutational fitness costs, and escape mechanisms can be used to identify immunogenic yet constrained viral regions and immune-relevant natural HIV sequence variation within them. For example, conserved epitopes and their common variants that retain intracellular processing and HLA binding ability might be considered immunogens, albeit with some caution (25). In particular, comparative analyses of HLA footprints in immunogenetically distinct host populations, wherein the same HIV subtype circulates, can illuminate the extent to which viral immunogenic regions and escape pathways are universal versus host population specific, thus potentially informing the design of universal and geographically tailored vaccine strategies.

Toward this goal, we compare HLA-associated HIV footprints in Gag and Pol in two large North American populations: the immunogenetically distinctive Mexican mestizo population, which features a mixture of Caucasian and Amerindian HLA alleles (20, 26–29), and the population of Canada/United States. Our study thus represents a unique opportunity to investigate the impact of host immunogenetics on HLA-associated adaptation in geographically proximal HIV subtype B epidemics. The present study significantly extends a preliminary study of HIV Pol adaptation by our group (20) by increasing cohort size by more than 5-fold, performing all adaptation analyses at HLA subtype-level resolution, and additionally analyzing Gag. As such, it represents the largest comparative study to date of differential HIV adaptation to HLA across human populations. Gag and Pol were studied because these proteins are rich in conserved epitopes where escape can be fitness costly (30–32) and where responses to these epitopes are associated with superior viremia control (7, 12, 14, 33–35). Overall, our results confirm that adaptation of HIV to HLA in Mexico, like in other global populations, occurs along broadly predictable pathways. HLA footprints observed in Mexico include canonical adaptation pathways described in many other populations (e.g., B*57 Gag-T242N [36] and B*51 RT-135X [11]) as well as novel pathways attributable to the unique HLA distribution of Mesoamerican peoples (e.g., Gag A*02:06-F44Y, B*39:02-E319D, and A*68:03-K436R; PR B*39:06-V15I and B*39:02-K70R; RT B*39:02-E79D, A*68:03-R103K, and B*35:12-P294S/T). Of note, however, HLA-associated HIV footprints in Mexico were fewer overall, and their strengths of selection significantly weaker, than those in Canada/United States, raising the intriguing hypothesis that HLA-restricted immune responses to HIV in Mexico are less potent, and/or HIV mutational escape pathways somewhat less consistent, than those in other populations.

## RESULTS

### Cohort description.

We studied two cohorts of antiretroviral-naive, HIV-1 subtype B chronically infected individuals from Mexico (*n* = 1,612) and Canada/United States (*n* = 1,641). Both cohorts were predominantly male (Mexico, 78.5%; Canada/United States, 85.1%). Median age at enrollment was 30 (interquartile range [IQR], 24 to 38) years in Mexico and 37 (32 to 44) years in Canada/United States. The median plasma viral load was 4.75 (IQR, 4.18 to 5.27) log_10_ RNA copies/ml in Mexico and 4.98 (4.55 to 5.46) log_10_ RNA copies/ml in Canada/United States. Median CD4^+^ T-cell counts were 311 (IQR, 121 to 519) cells/μl in Mexico and 260 (110 to 400) cells/μl in Canada/United States. Calendar years of enrollment were 2000 to 2014 in Mexico and 1996 to 2004 in Canada/United States.

### Gag and PR-RT sequence diversity in Mexico and Canada/United States.

**FIG 1:**
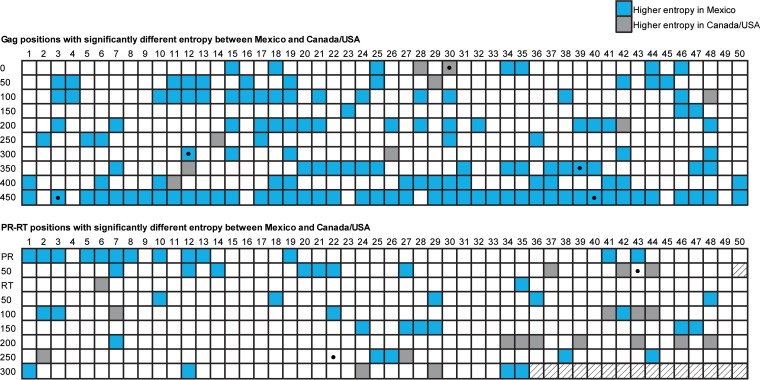
Entropy differences in Gag and PR-RT on HIV from Mexico and Canada/United States. Comparison of Shannon entropy scores at each HIV position between cohorts. Each box represents an HIV codon. The top panel shows the 500 Gag positions; the bottom panel shows the 99 protease (PR) and the first 335 reverse transcriptase (RT) codons. Positions with significantly different Shannon entropies between Mexico and Canada/United States (*P* < 0.001) are colored blue for positions with higher entropy in Mexico and gray for positions with higher entropy in Canada/United States. Black dots denote positions with different consensus amino acids between cohorts. The complete list of entropy values for Gag and PR-RT is available in Tables S4 and S5 in the supplemental material, respectively.

**FIG 2:**
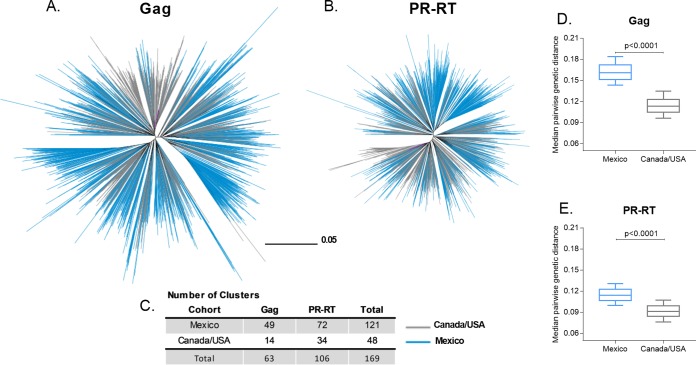
Genetic diversity of HIV-1 subtype B Gag and PR-RT from Mexico and Canada/United States. Unrooted maximum likelihood phylogenetic trees inferred from Gag (*n* = 2,771) (A) and PR-RT (B) sequences (*n* = 3,084), drawn on the same genetic distance scale. Branch color indicates the cohort to which the sequence belongs. Purple branches denote the HXB2 subtype B reference sequence. (C) Number of clusters defined by within-cluster patristic distances of ≤1.5% and bootstrap support of ≥90% in each tree. Median pairwise genetic distance comparison between Mexico (blue box) and Canada/United States (gray box) for Gag (D) and PR-RT (E).

We first assessed HIV subtype B diversity and phylogenetic relationships between our cohorts. Gag and PR-RT sequences were available for 1,450 and 1,529 individuals, respectively, in Mexico, and 1,320 and 1,555 individuals, respectively, in Canada/United States. Cohort-specific consensus amino acid sequences differed at only 5 (of 500; 1%) Gag codons (positions 30, 312, 389, 403, and 490) and 2 (of 434; 0.5%) PR-RT codons (PR 93 and RT 272). Overall, Gag amino acid entropy was significantly higher in Mexico than Canada/United States (median 0.056 versus 0.026), respectively; *P* < 0.0001. In particular, 38.2% (191/500) of Gag codons showed significantly higher entropy in the Mexican cohort, while only 4% (20/500) showed higher entropy in the Canada/United States cohort (Fig. 1; also see Table S4 in the supplemental material). In contrast, PR-RT entropy in Mexico was comparable to that of Canada/United States both overall (median 0.022 versus 0.031, respectively; *P* = 0.08) and in terms of the proportion of codons with significantly higher entropy in one cohort versus the other (∼17 to 18%) (Fig. 1 and Table S5). We next inferred phylogenies from Gag and PR-RT nucleotide alignments (Fig. 2). As expected, overall Gag sequence diversity exceeded that of PR-RT. Also as expected, given their proximity on the North American continent, Mexico and Canada/United States sequences were quite intermixed in their phylogenies (fixation indices [F_ST_] were very low for both Gag [0] and PR-RT [0.006]). Moreover, while no statistically supported clusters containing sequences from both cohorts were found at a genetic distance of ≤1.5% and bootstrap support of ≥90%, increasing the distance threshold to 4.5% yielded 3 clusters for Gag and 4 in PR-RT containing sequences from both cohorts with 90% bootstrap support. On average, Mexican Gag and PR-RT sequences exhibited higher median patristic distances compared to those of Canada/United States sequences (Gag, 0.1612 and 0.1132; PR-RT, 0.1145 and 0.0912 for Mexico and Canada/United States, respectively; *P* < 0.0001 in both cases). Overall, results support an interlinked North American HIV-1 subtype B epidemic where overall nucleotide diversity is higher in Mexico.

### HLA allelic frequency comparison between Mexico and Canada/United States.

**FIG 3:**
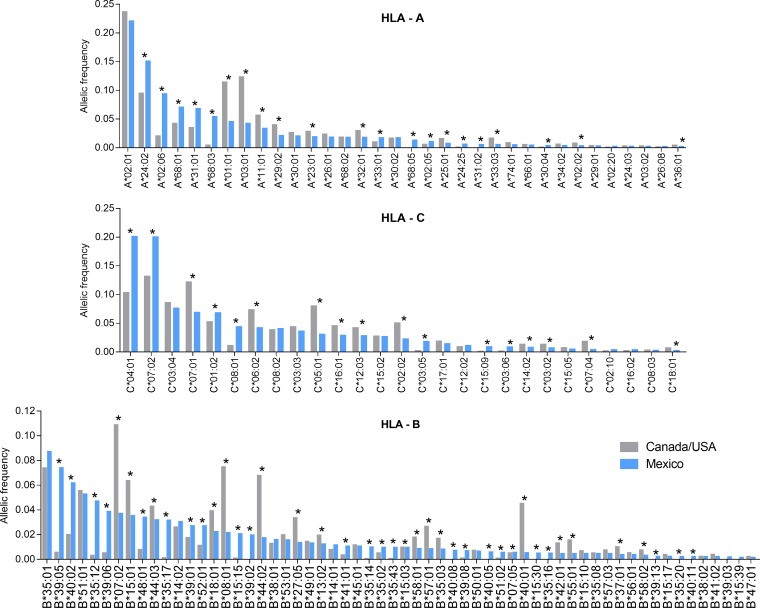
Differences in HLA frequencies between Mexico and Canada/United States. Comparisons of HLA frequencies between Mexico (*n* = 1,612; blue bars) and Canada/United States (*n* = 1,641; gray bars). HLA alleles are ordered by descending frequency in the Mexican cohort. HLA alleles with less than 0.1% frequency in the Mexican cohort are not shown. The complete list of comparisons can be found in Table S6 in the supplemental material. *, *P* < 0.05 and q < 0.2.

A total of 252 HLA class I alleles, defined at subtype-level resolution, were observed (Fig. 3 and Table S6). Of these, 140 were observed in both cohorts, 67 were observed exclusively in Mexico, and 45 were exclusively in Canada/United States. In Mexico, the most frequent HLA alleles were A*02:01, A*24:02, and A*02:06 for the A locus; B*35:01, B*39:05, and B*40:02 for the B locus; and C*04:01, C*07:02, and C*03:04 for the C locus. In Canada/United States, these were A*02:01, A*03:01, and A*01:01 for the A locus; B*07:02, B*35:01, and B*08:01 for the B locus; and C*07:02, C*07:01, and C*04:01 for the C locus (Fig. 3). Of the 252 HLA alleles observed, 86 (22 HLA-A, 46 HLA-B, and 18 HLA-C) differed significantly (*P* < 0.05 and q < 0.2) in frequency between Mexico and Canada/United States (Fig. 3) (note that when HLA frequencies were computed separately by cohort, 81 alleles differed significantly in frequency between Canada and Mexico, 77 between the United States and Mexico, and only 57 between Canada and the United States [Table S6]). Of these 86 HLA alleles, 41 were significantly more frequent in Mexico than in Canada/United States; these included A*24:02, A*02:06, A*68:01, A*31:01, A*68:03, B*39:05, B*40:02, B*39:06, C*04:01, C*07:02, and C*01:02 alleles, which are enriched in mestizo and Amerindian populations (26, 27, 29). Consistent with previous reports (20, 37), most canonical protective HLA alleles (37) were enriched in the Canada/United States cohort compared to that from Mexico (e.g., B*57:01, B*58:01, B*27:05, B*13:02, B*42:01, B*44:03, A*25:01, and A*32:01). Overall, these results reveal marked immunogenetic differences between Mexico and Canada/United States cohorts.

### Differential HLA footprints on HIV Gag and PR-RT in Mexico and Canada/United States.

**FIG 4:**
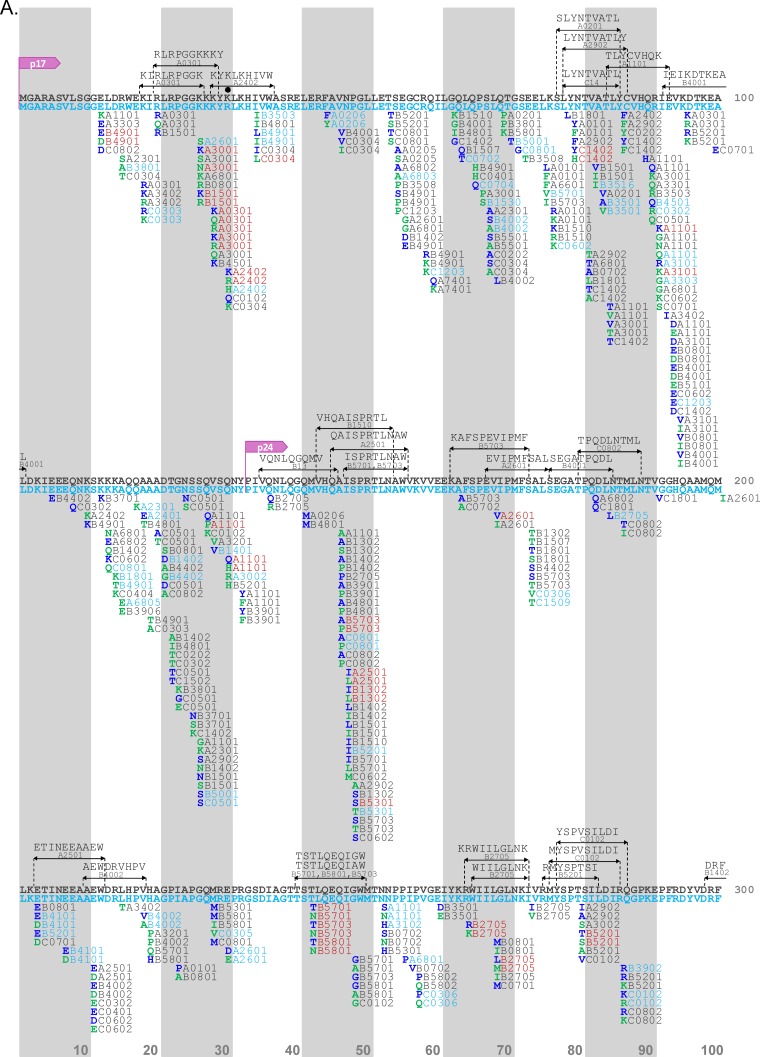

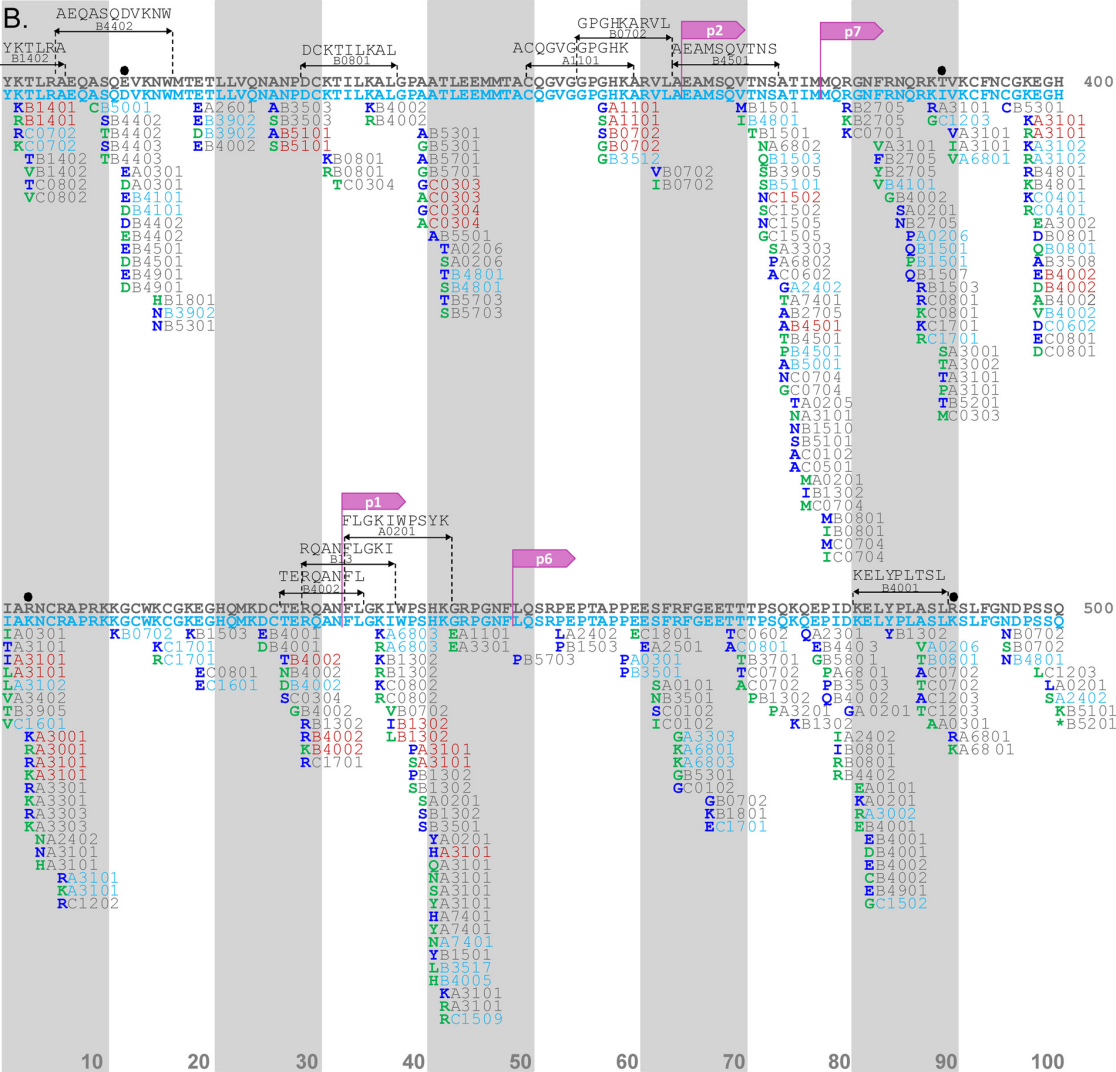
Comparative Gag immune escape map for Mexico and Canada/United States. The escape map shows the location of HLA-associated polymorphisms in HIV-1 subtype B *gag* sequences from Mexico (*n* = 1,450) and Canada/United States (*n* = 1,320) at *P* < 0.05 and q < 0.2. The reference sequence represents the cohort-specific consensus sequence: blue for Mexico and gray for Canada/United States. Black dots denote codons where the consensus sequence differs between cohorts. (A) Gag positions 1 to 300; (B) Gag positions 301 to 500. One hundred amino acids are displayed per line; vertical bars separate blocks of 10 amino acids. Adapted amino acids are shown in boldface green letters and nonadapted amino acids are shown in boldface blue letters, along with their restricting HLA allele(s) (blue for Mexico, gray for Canada/United States, and red for shared associations observed at a q value of <0.2 in both cohorts). Published optimal epitopes harboring HLA-polymorphism associations are shown above the consensus sequences in black. The complete list of associations can be found in Tables S7 and S8 in the supplemental material.

**FIG 5:**
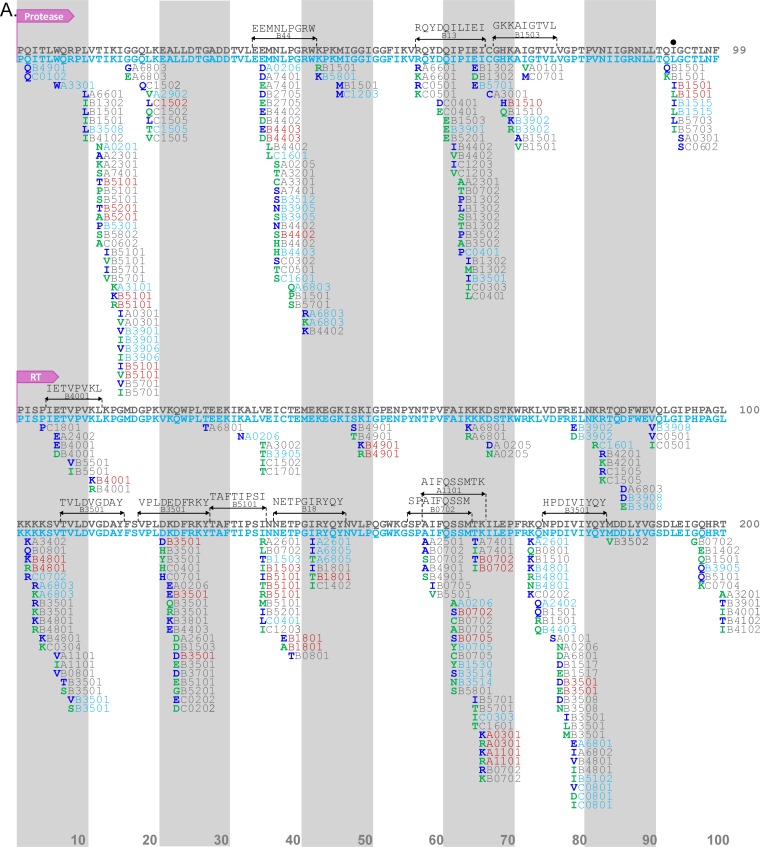

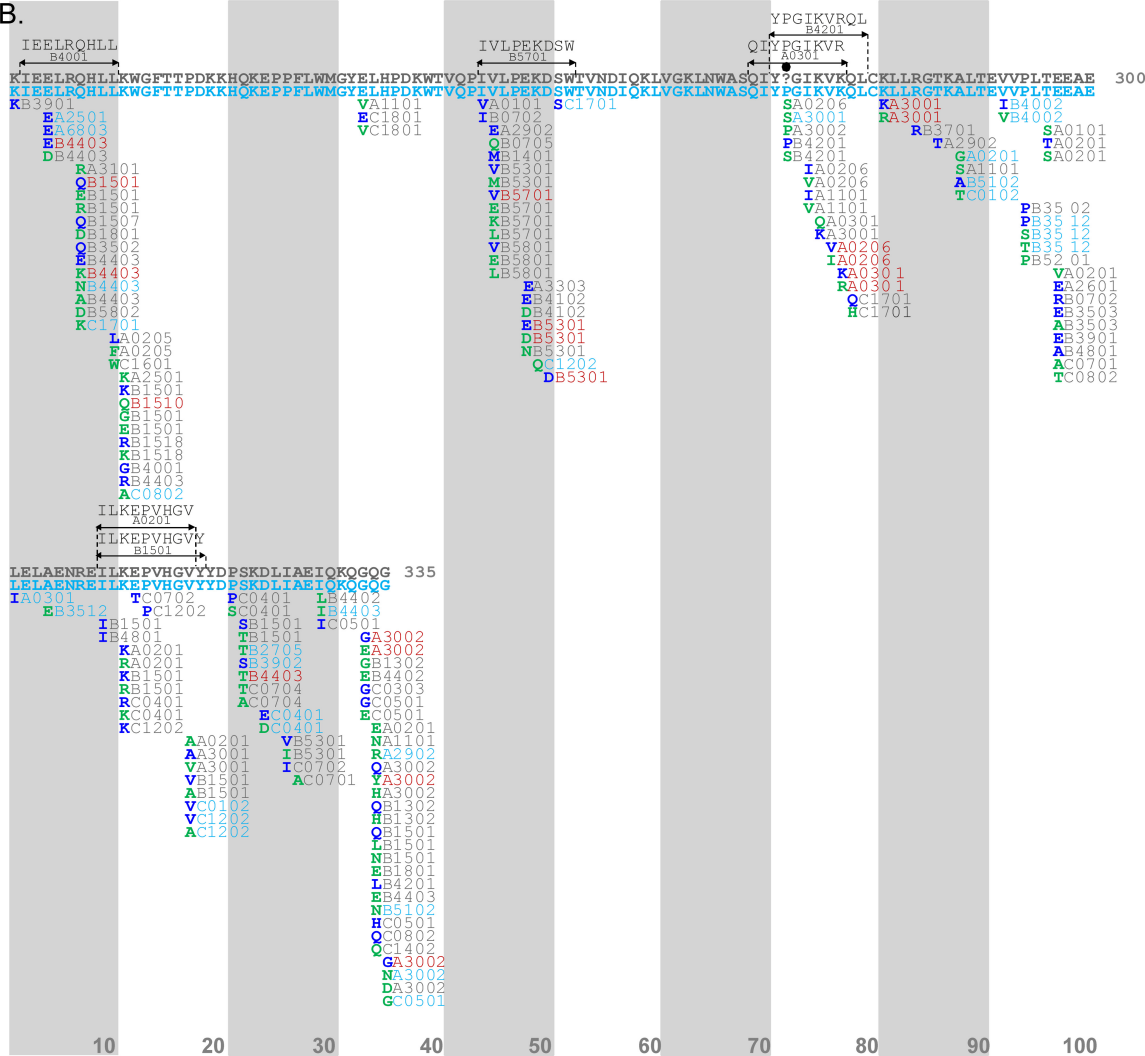
Comparative PR-RT immune escape map for Mexico and Canada/United States. The escape map shows the location of HLA-associated polymorphisms in HIV-1 subtype B PR-RT sequences from Mexico (*n* = 1,529) and Canada/United States (*n* = 1,555). (A) PR positions 1 to 99 and RT positions 1 to 200; (B) RT positions 201 to 335. Features of this map are the same as those seen in Fig. 4. The complete list of associations can be found in Tables S7 and S8 in the supplemental material.

Given the marked immunogenetic differences in neighboring North American populations, we hypothesized that HLA-associated polymorphisms also would differ between them. We identified HAPs in the Mexican and Canada/United States data sets using established methods (14) and constructed HIV immune escape maps showing HAPs identified in one or both cohorts at a q value of <0.2 (Fig. 4 and 5 and Tables S7 and S8). In the Mexico data set, we identified a total of 201 HAPs (108 adapted; 93 nonadapted) that occurred at 95 (of 500; 19%) Gag codons that were restricted by 66 HLA alleles. In the Canada/United States data set, we identified a total of 534 HAPs, significantly more than those in the Mexico data set (*P* < 0.0001), at 166 (32.3%) Gag codons that were restricted by 77 HLA alleles. Overall, these summed to 662 unique HAPs identified in Gag, of which 73 (11.02%) (35 adapted and 38 nonadapted, occurring at 26 Gag codons) were identified in both cohorts, 128 were identified only in Mexico, and 461 were identified only in Canada/United States at the predefined statistical threshold of a q value of <0.2 (Fig. 4). Consistent with previous reports (7), the total proportion of p24^Gag^ codons harboring HAPs was lower than that of the rest of Gag, both in Mexico (12.1% [28/231] for p24 versus 24.5% [66/269] for other Gag proteins) and in Canada/United States (19.0% [44/231] versus 45.4% [122/269]). This is expected given p24^Gag^'s high overall sequence conservation (>50% of codons are 99.5% to 100% conserved, which precludes identification of HLA associations at these positions). However, if one instead uses the total number of variable codons as the denominator, p24 ranks among the richest areas in the HIV proteome for HLA associations (7, 38) which is true also for Mexico.

In PR-RT, we identified 157 HAPs (78 adapted and 79 nonadapted) restricted by 58 HLA alleles, occurring at 70 codons, in the Mexico data set. In the Canada/United States data set we found 371 HAPs (201 adapted and 170 nonadapted) restricted by 78 HLA alleles, occurring at 105 codons (again significantly more than those in Mexico; *P* = 0.0039) (Fig. 5). Overall, these summed to 470 unique HAPs identified in PR-RT, of which 58 (12.3%) were identified in both cohorts, 99 were identified only in Mexico, and 313 were identified only in Canada/United States at the predefined statistical threshold of a q value of <0.2. Of note, all 7 codons (5 in Gag and 2 in PR-RT) where the consensus amino acid differed between cohorts showed evidence of HLA selection.

As noted above, a substantial fraction of HAPs was observed in both cohorts at a q value of <0.2, further supporting the existence of universal HLA-associated escape pathways across human populations globally. These shared associations included canonical CTL escape pathways within epitopes restricted by protective HLA class I alleles, including B*57:01/B*57:03-Gag-T242N (within the TW10 epitope restricted by these alleles), B*27:05-Gag-R264K and L268M (within the B*27-restricted KK10 epitope) (39–43), and B*51:01-RT-I135T (within the B*51-restricted TI8 epitope) (43, 44), as well as previously described HAPs within optimal epitopes (7), including A*03:01-K28Q/R, A*24:02-K30R, B*57:03-A146P, B*14:01-K302R (45), B*07:02-S357G (46), and B*40:02-R429K (47) in Gag, B*44:03-E35D (48) in protease, and A*11:01-K166R (49), B*35:01-D177E (50), and A*03:01-K277R (51) in RT (Fig. 4 and 5).

However, we also observed a substantial number of novel HAPs in Mexico that were not within previously described optimal epitopes. Moreover, these novel HAPs tended to be associated with Amerindian HLA alleles. Examples include A*02:06-F44Y, B*35:16-X82I, A*26:01-D230E, B*39:02-R286X, B*39:02-N315X, B*39:02-E319D, B*35:12-X357G, A*02:06-P386X, and A*68:03-K436R in Gag; B*39:06-V15I in protease; and A*02:06-I274V and A*02:06-V276I in RT.

Taken together, HLA footprints in Mexico include both canonical HIV escape pathways shared across global populations as well as novel HAPs restricted by HLA alleles typically found in Amerindian or mestizo populations.

### HLA footprints on HIV in Mexico are scarcer and weaker than those in Canada/United States.

**FIG 6:**
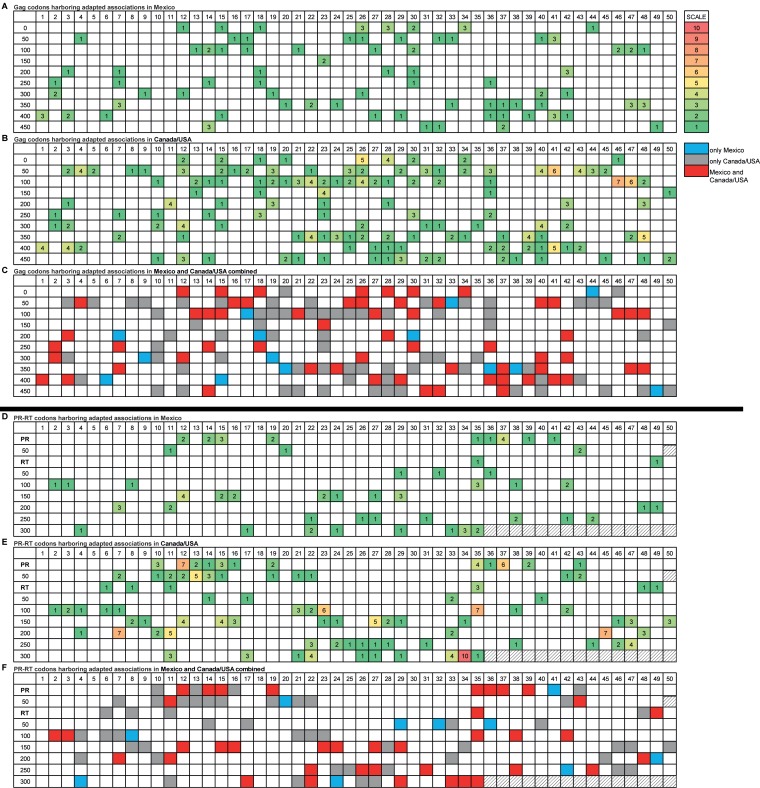
Distribution of Gag and PR-RT HIV codons harboring adapted HLA associations in Mexico and Canada/United States. (A to C) Gag codons; (D to F) PR-RT codons. Panels A and D show HIV codons harboring adapted associations in Mexico, and panels B and E show HIV codons harboring adapted associations in Canada/United States. The number in each box corresponds to the number of HLA-adapted associations observed at that specific position. Panels C and F provide a merged map showing HLA-adapted associations present in Mexico only (blue), Canada/United States only (gray), and in both cohorts (red).

A particularly striking observation from our analysis was the overall lower number of HLA footprints in Mexico than in Canada/United States, despite cohorts being of comparable sizes. For example, considering only HIV codons at which adapted associations were identified with one or more HLA alleles, not only did the Mexican cohort exhibit fewer such codons in Gag than the Canada/United States cohort (108 at 75 Gag codons versus 273 at 133 Gag codons, respectively; *P* < 0.0001) but Mexico also exhibited a lower number of adapted associations per codon (up to 3 HLA alleles per codon versus up to 7 HLA alleles per codon in Canada/United States) (Fig. 6). The same was true when all HLA-associated codons (adapted and nonadapted) were analyzed (data not shown). Specifically, of all Gag codons harboring HLA-adapted associations, fewer than 10% were identified exclusively in Mexico, while the remainder were observed in both cohorts (41%) or in Canada/United States only (49%) (Fig. 6A to C). Similar results were observed for PR-RT (78 adapted associations at 49 positions in Mexico versus 201 adapted associations at 87 positions in Canada/United States; *P* < 0.0001), where 10% were exclusively observed in Mexico, 40% were observed in both cohorts, and 50% were observed in Canada/United States only (*P* < 0.0001) (Fig. 6D to F). As a result, the number of immunogenic zones (defined as consecutive HIV amino acids harboring an adapted HLA association) also differed markedly between cohorts: whereas stretches of up to 11 positions under HLA pressure were observed in Canada/United States (e.g., Gag 118 to 128), the longest such zone was only 3 amino acids for Mexico. Furthermore, where immunogenic zones did occur in Mexico, these tended to coincide with zones also identified in Canada/United States (e.g., Gag 146 to 148 and PR 35 to 37).

**FIG 7:**
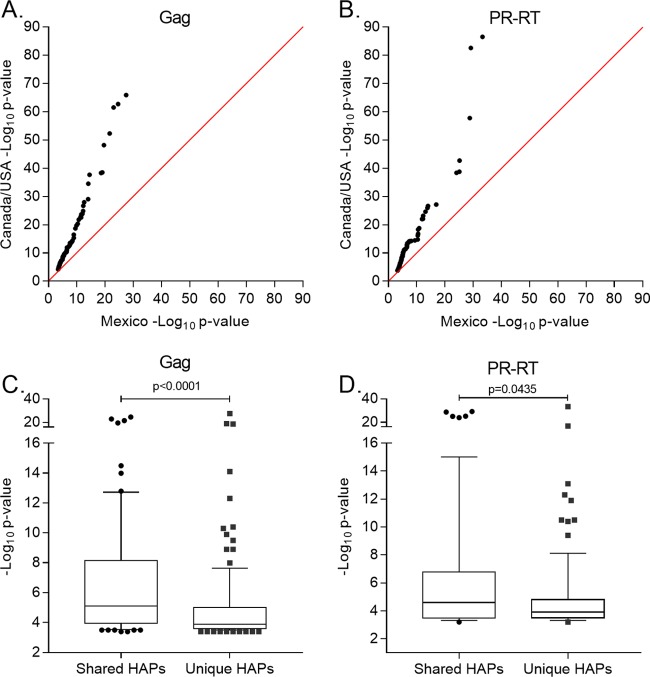
Comparison of HAP *P* value distributions between Mexico and Canada/United States. −Log_10_ *P* value transformations for the top 201 Gag HAPs found in Mexico and Canada/United States (A) and the top 157 PR-RT HAPs found in Mexico and Canada/United States (B) are shown. Transformed *P* values are ranked from smallest (least significant) to largest (most significant) in each cohort and plotted as paired observations. The red line represents the null expectation. (C and D) −Log_10_ *P* value distribution of shared versus unique HAPs observed in Mexico in Gag (C) and PR-RT (D).

The scarcer HLA footprint in Mexico likely is at least partially attributable to the higher HIV and HLA diversity in Mexico than that in Canada/United States (Fig. 1 to 3). This increases the total number of HLA-HIV pairwise comparisons required for Mexico, yielding a more stringent *P* value cutoff mapping to a q value of <0.2 for this cohort. Indeed, HAP identification required 726,206 HLA-HIV comparisons for Mexico compared to 592,677 for Canada/United States, such that a q value of <0.2 mapped to *P* < 10^−4^ in Mexico but *P* < 10^−3^ in Canada/United States (Tables S7 and S8). However, our observations are not solely explained by multiple-comparison correction. This is because, in addition to HLA footprints being scarcer overall, the statistical strengths of association between HLA alleles and HIV codons in Mexico also are weaker overall than those observed in Canada/United States. For example, a comparison of ranked −log_10_-transformed *P* values for the top 201 Gag and 157 PR-RT associations between cohorts (201 and 157, because these represented the total number of HAPs identified at a q value of <0.2 in Gag and PR-RT in Mexico) reveals that the Canada/United States one was always higher (i.e., more significant) than its corresponding Mexican one of the same ranking (Fig. 7A and B). This indicates that the strongest HLA footprints in Mexico overall are far weaker than the strongest HLA footprints in Canada/United States. Moreover, when analyzing only the −log_10_ *P* value distribution of HAPs identified in Mexico, we observed lower (less significant) overall values for those identified exclusively in Mexico compared to those shared with Canada/United States for both Gag (*P* < 0.0001) and PR-RT (*P* = 0.0435) (Fig. 7C and D). Thus, the strength of association between HLA alleles and HIV codons appears to be inherently weaker in Mexico than in Canada/United States, and of the HLA footprints that are detectable in Mexico, the strongest tend to be ones that are already known, whereas the novel HAPs restricted by unique mestizo HLA alleles tend to be even weaker.

**FIG 8:**
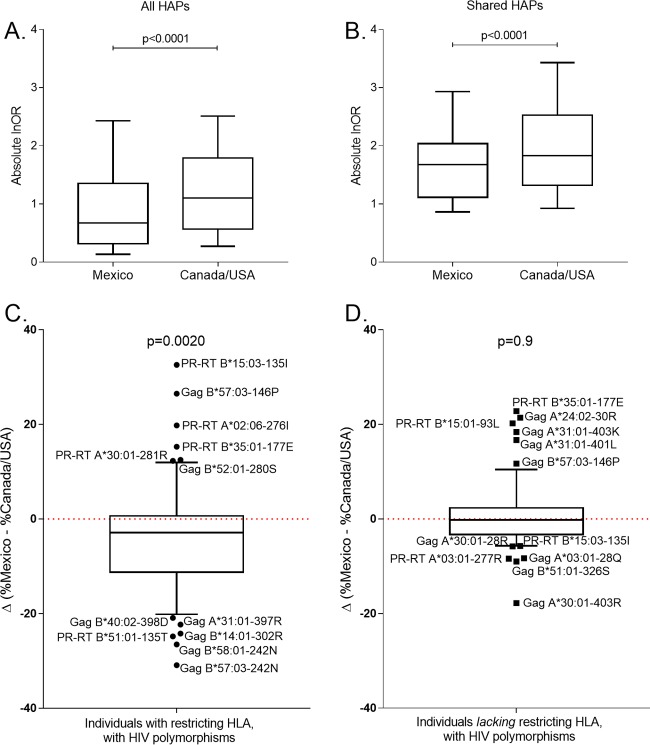
Weaker HLA-associated footprint in Mexico compared to that in Canada/United States. (A) Pairwise comparisons of the absolute log-transformed odds ratios (Absolute lnOR) for all HAPs identified in Mexico and/or Canada/United States that were restricted by HLA alleles observed in a minimum of 10 individuals in both cohorts (*n* = 995). (B) Same as panel A but restricted to shared HAPs (i.e., those identified in both cohorts at a q value of <0.2 in the original analysis; *n* = 131). Results support a significantly weaker HLA-associated footprint in Mexico than in Canada/United States. (C) The difference in the percentage of persons expressing the restricting HLA and harboring the relevant adapted HIV variant in Mexico versus Canada/United States, where a negative value indicates the variant is less frequently found among HLA-expressing persons in Mexico. The horizontal line denotes the median, box edges denote the 25th and 75th percentiles, whiskers denote 10th to 90th percentiles, and individual outliers are labeled. The *P* value is derived from a Wilcoxon matched-pairs test applied to the corresponding variant frequencies between cohorts. (D) The difference in the percentage of persons lacking the restricting HLA and harboring the relevant adapted HIV variant in Mexico versus Canada/United States. The *P* value is derived from a Wilcoxon matched-pairs test applied to the corresponding variant frequencies between cohorts.

We extended this analysis by comparing HAP selection strength across cohorts in a pairwise fashion. To do this, we took the union of all HAPs identified in either Mexico and/or Canada/United States that were restricted by HLA alleles observed in a minimum of 10 individuals in both cohorts (it is not possible to compare strengths of selection of HAPs restricted by HLA alleles that are not observed, or only very rarely observed, in a given cohort). This yielded a total of 995 HAPs for analysis (Table S9). Pairwise comparison of the absolute log-transformed odds ratios (absolute lnOR) of selection for each HAP across the two cohorts revealed statistically significantly higher values for Canada/United States (median, 1.1; IQR, 0.57 to 1.8) than in Mexico (median, 0.67; IQR, 0.32 to 1.4) by Wilcoxon matched-pairs test (*P* < 0.0001) (Fig. 8A). These results remained consistent upon stratification by HIV protein and when analyses were restricted to unique HLA-HIV codon pairs (to avoid double counting of adapted and nonadapted associations at the same codon) (*P* < 0.0001 and data not shown). Similarly, results remained consistent when the analysis was restricted to shared HAPs (a total of 73 HAPs in Gag and 58 in PR-RT were observed in both Mexico and Canada/United States and were restricted by HLA alleles observed in at least 10 individuals in both cohorts). Again, the absolute log-transformed odds ratios of selection of these HAPs were significantly higher in Canada/United States (median, 1.8; IQR, 1.3 to 2.5) than in Mexico (median, 1.7; IQR, 1.1 to 2.0) overall (*P* < 0.0001 by Wilcoxon matched-pairs test) (Fig. 8B). These results remained consistent upon stratification by HIV protein and when analysis was restricted to unique HLA-HIV codon pairs (*P* values of <0.05 in all cases; data not shown). Thus, our observations indicate that, on a per-HAP basis, HLA footprints on HIV in Mexico are, on average, significantly weaker than those in Canada/United States.

### Scarcer and weaker HLA footprints on HIV in Mexico are not explained by challenges associated with HLA typing in this population.

HLA class I typing of highly admixed human populations can be challenging due to elevated genetic diversity. To rule out ambiguous and/or imputed HLA calls as possible contributors to our observation of scarcer and weaker HLA footprints on HIV in Mexico, we repeated all analyses, excluding 255 HLA loci for which the original types were ambiguous in the Mexican cohort (these included 222 [92 HLA-A, 92 HLA-B, and 39 HLA-C] loci with phase ambiguities and 33 HLA-A or -C types that had been imputed due to failed amplification/sequencing). Results were entirely consistent with those of the original analysis (Fig. S1). First, the number and location of HLA-associated polymorphisms identified in Mexico were >80% consistent with those reported in the original manuscript (∼20% discordance is expected given our use of a q value correction for multiple testing; at a q value of <0.2, we expect ∼20% of identified associations to be false positives) (Fig. S1A). Second, the *P* values of HLA-associated polymorphisms identified in the original and revised analyses are highly concordant (Spearman's *R* of 0.825, *P* < 0.0001) (Fig. S1B). Most importantly, results of the reanalysis fully corroborate our original observations of significantly fewer and weaker HLA-associated footprints in Mexico than in Canada/United States (Fig. S1C to H). These results indicate that the scarcer and weaker HLA footprints on HIV in Mexico are not explained by challenges associated with HLA typing in this population.

### Exploring reasons for weaker HLA selection on HIV in Mexico.

Two possibilities, which are not necessarily mutually exclusive, could explain the scarcer and weaker HLA footprints on HIV in Mexico. The first is that HLA-restricted CTL responses on a given HIV codon are weaker, and/or the virus-preferred escape pathways less predictable, in Mexico than elsewhere. Therefore, for each shared adapted HAP restricted by an HLA allele observed in a minimum of 10 individuals in both cohorts, we compared its prevalence in persons expressing the restricting HLA with the hypothesis that if HLA-mediated selection was weaker or less predictable in Mexico, polymorphism prevalence in HLA-expressing persons would be lower overall in Mexico than in Canada/United States. The second possibility is that HIV sequences circulating in Mexico already harbor a high burden of HLA-adapted mutations, thus reducing the power to detect further enrichment of these variants in persons expressing the restricting HLA. We therefore also compared the prevalence of each shared adapted HAP in persons lacking the restricting HLA with the hypothesis that if circulating adaptation was higher in Mexico, these values would be higher overall in Mexico than in Canada/United States.

We take the well-described B*51:01-RT I135T mutation as an example. While it is identified in both cohorts, its lnOR of selection is 1.23 in Mexico versus 2.40 in Canada/United States, a statistically significant difference (phylogenetically informed logistic regression test *P* value of 8.4 × 10^−7^). Computing the frequencies of RT I135T in HLA-B*51:01- and non-B*51:01-expressing individuals across cohorts, we note that less than 50% (68/143) of B*51:01-expressing Mexican individuals harbor 135T compared to nearly two-thirds of B*51:01-expressing individuals in Canada/United States (105/144) (*P* < 0.0001 by Fisher's exact test). This suggests that the weaker association between B*51:01 and RT-135T in Mexico is because B*51-restricted CTLs in this population do not respond as strongly or frequently to the TI8 epitope (or that HIV does not escape as reproducibly via selection of T at this position in response to this pressure) as the HIV subtype B-infected populations to the north. On the other hand, the prevalence of RT-I135T in persons lacking B*51:01 is approximately 20% in both cohorts (282/1,350 and 256/1,330 for Mexico and Canada/United States, respectively; *P* = 0.3 by Fisher's exact test), suggesting that the weaker association between B*51:01 and RT-135T in Mexico is not attributable to elevated frequencies of circulating HIV harboring this mutation.

When we applied these analyses to all 61 adapted shared HAPs, we observed that, overall, the proportion of individuals expressing the restricting HLA and harboring the adapted HIV variant was a median of 2.9% lower in Mexico compared than in Canada/United States (IQR, −11.25 to 0.65%; *P* = 0.0020 by Wilcoxon matched-pairs test) (Fig. 8C), supporting weaker HLA-mediated selection in the latter region. There was, nevertheless, a wide distribution in the data, with certain polymorphisms observed more frequently in one cohort than in the other. For example, among the HAPs that were observed more frequently among HLA-expressing persons in Canada/United States than in Mexico were well-characterized escape mutations restricted by protective HLA alleles, including Gag B*57:03-242N (with 45.5% [5/11] of Mexican B*57:03s selecting for N and 75% [18/24] of Canada/United States B*57:03s), Gag B*58:01-242N (60% [12/20] and 86.5% [44/51] for Mexico and Canada/United States, respectively), and B*51:01-RT135T (described above). In contrast, a minority of HAPs were observed more frequently among HLA-expressing persons in Mexico, including Gag B*57:03-146P (observed in 81.8% versus 54.8% of B*57:03-expressing persons in Mexico compared to Canada/United States).

On the other hand, the frequencies of HAPs among individuals lacking the relevant HLA allele were not overall significantly different between cohorts (*P* = 0.9 by Wilcoxon matched-pairs test), although we did note examples of specific HIV polymorphisms restricted by relatively common HLA alleles in Mexico that were significantly more prevalent in circulation in Mexico than in Canada/United States (e.g., Gag A*24:02-30R, circulating frequency of 54% in Mexico and 33% in Canada/United States; A*31:01-403K, 58% in Mexico versus 37% in Canada/United States) (*P* < 0.0001 for both HAPs by Fisher's exact test) (Fig. 8D). Taken together, our observations suggest that even though preadaptation of HIV to certain common HLA alleles is observed in Mexico, the sparser and weaker HLA footprints on HIV in Mexico overall are more attributable to weaker CTL pressure (and/or less reproducible escape) in this population than in those to the north.

### Some HLA footprints are stronger in Mexico than in Canada/United States.

**FIG 9:**
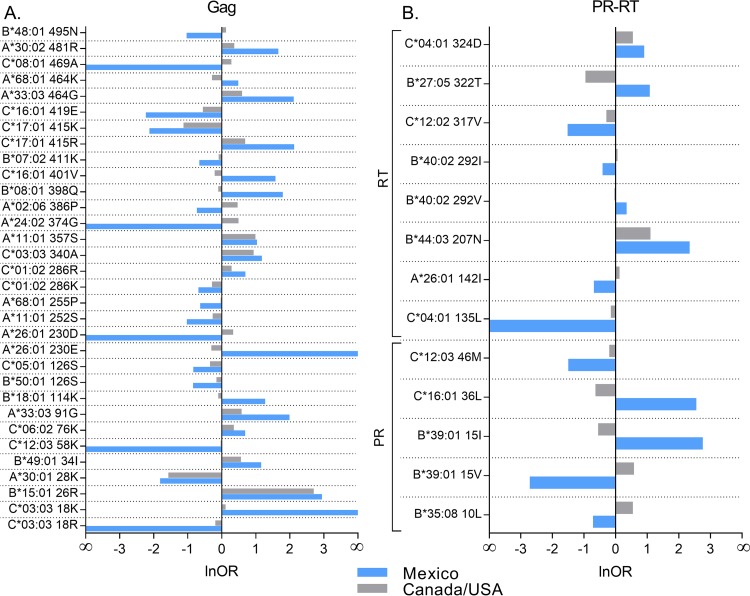
HLA-associated HIV polymorphisms showing stronger HLA-associated selection in Mexico than in Canada/United States. We took all HLA-associated HIV polymorphisms (HAPs) identified in Mexico that were restricted by HLA alleles observed in a minimum of 10 individuals in Canada/United States and applied a phylogenetically corrected logistic regression test to compare their strengths of association across cohorts. HAPs displaying significantly stronger HLA-associated selection in Mexico (*P* < 0.05, q < 0.2) in Gag (A) and PR-RT (B) are shown. The complete list of comparisons is available in Table S9 in the supplemental material.

Although our results reveal an overall weaker HLA footprint on HIV in Mexico than Canada/United States, nevertheless there are some exceptions. To identify these, we took all HAPs identified in Mexico that were restricted by HLA alleles observed in a minimum of 10 individuals in Canada/United States and applied a phylogenetically corrected logistic regression test to compare their strengths of association across cohorts. Of the 233 HAPs analyzed (137 in Gag and 96 in PR-RT), 45 (19.31%) exhibited significantly stronger selection, as measured by higher absolute lnOR, in Mexico than in Canada/United States (all *P* < 0.05, q < 0.2) (Fig. 9). Among these were A*24:02-374G, A*02:06-386P, B*15:01-126S, and B*08:01-398Q in Gag, B*39:01-15 in PR, and C*04:01-324D in RT, suggesting that these HLA alleles mount stronger and/or more consistent immune pressure on these HIV sites in the Mexican population than those farther north. Of note, we found no examples of Mexican HAPs that exhibited diametrically opposed selection in Canada/United States (that is, where the significant HIV-adapted form for a given HLA allele in Mexico represented the significant nonadapted form in Canada/United States or vice versa).

## DISCUSSION

Although HLA-associated polymorphisms in HIV are being elucidated increasingly in global populations (14, 16, 17, 20, 52–54), our study is notable because it compares HLA footprints identified in Mexico, which comprises a highly genetically admixed and, thus, immunogenetically unique mestizo population that includes mainly Amerindian and European but also African and East Asian ancestry components (26, 27), to those in HIV subtype B-infected populations to the north (16, 52–55), allowing us to investigate the impact of host immunogenetics on HIV adaptation in neighboring epidemics. We observed that HLA footprints on HIV in Mexico include well-known associations, such as those restricted by protective HLA class I alleles (e.g., Gag B*57:01-T242N, Gag B*27:05-R264K/L268M [39–43], and RT B*51:01-I135T [43, 44]), as well as novel associations restricted by HLA alleles enriched in mestizo populations (e.g., B*39:02, B*39:05, B*35:12, B*35:14, A*02:06, and A*68:03). Our results strengthen the growing body of evidence supporting both universal and region-specific immune escape pathways attributable to host population immunogenetic composition (17, 20).

An unanticipated observation was that HLA footprints in Mexico overall were sparser (we observed 61% fewer HAPs in Mexico than in Canada/United States) and, on average, were weaker (in terms of lower odds ratios and higher *P* values) than those in Canada/United States. While the higher HLA and HIV diversity in Mexico reduces statistical power to identify associations to some extent, in part because of the need to correct for a larger number of HLA/HIV comparisons, this is not the sole explanation. Similarly, challenges associated with HLA typing of the highly genetically admixed Mexican population was also ruled out as an explanation in detailed sensitivity analyses (see Fig. S1 in the supplemental material). Rather, exploration of our data suggested that the sparse HLA footprint on HIV in Mexico is not due to widespread viral preadaptation (25) to HLA class I alleles (although individual exceptions were noted) but rather were due to weaker or less frequent HLA-restricted CTL responses on HIV, and/or less reproducible viral escape from these responses, in the Mexican population. The canonical B*51:01-RT-I135T association provides an example. Despite similar HLA-B*51:01 and RT codon 135 frequencies across cohorts, this association is significantly weaker in Mexico than in Canada/United States. The observation that RT-135T is not as prevalent among B*51:01-expressing persons in Mexico (47.6%) than in those in Canada/United States (72.4%) (note that the same is true when one considers all RT codon 135 variants, i.e., RT-I135X, which occur in 72% of B*51:01-expressing persons in Mexico and 94% in Canada/United States) while the frequency of RT-135T is comparable (∼20%) in individuals lacking B*51:01 across cohorts suggests that the weaker B*51 footprint on this HIV codon in Mexico is due to weaker B*51:01-mediated immune pressure (and/or less reproducible viral escape) in Mexico than in Canada/United States and is not due to accumulation of this variant in circulation. This observation contrasts with those for Japan, where a similarly weak association between B*51:01- and RT-I135T at the population level in this region instead is attributable to the accumulation of this variant in circulation to the point that it has become the consensus (11, 17).

Furthermore, a much larger fraction of HAPs identified in Mexico constituted associations shared with Canada/United States than vice versa. For example, 36.6% (131/358) of HAPs identified in Mexico were shared with Canada/United States (that is, only 63.4% were specific to Mexico), whereas of the 905 associations identified in Canada/United States, 774 (85.5%) were exclusive to this region and only 131 (14.5%) were shared with Mexico. In other words, the footprints left on HIV by typical Mexican (i.e., mestizo) alleles were fewer than expected given the size of the cohort. Moreover, absolute −log_10_ *P* values and lnOR of HAP unique to Mexico were significantly weaker than those shared with Canada/United States. Finally, it is worth noting that the stronger HLA footprint in Canada/United States than in Mexico is not likely to be driven by the lower frequencies of canonical protective alleles in the latter region. Support for this is provided by our analyses comparing the proportion of individuals expressing the restricting HLA and harboring the escape variant of interest, which are agnostic to HLA frequency. For example, Gag-242N was observed in 75% (18/24) of B*57:03-expressing persons in Canada/United States but only 45.5% (5/11) in Mexico and Canada/United States, suggesting weaker selection strength in Mexico independent of B*57:03 prevalence. Together, our observations suggest that population-level HLA pressures on HIV, in particular those attributable to HLAs enriched among mestizos, are inherently weaker in Mexico than in populations to the north.

Before proposing possible underlying mechanisms, some limitations and potential confounders merit mention. First, cohort CD4 count distributions suggest more advanced infection in the Canada/United States than in the Mexico cohort; we therefore cannot rule out a longer time for within-host escape mutations to accumulate (and, thus, enhanced ability to detect them) in the former. However, given that the majority of escape occurs in the initial year or two following infection (56–59), that escape is sufficiently frequent and reproducible to be detected at the population level as early as 6 months postinfection (60), and that both study cohorts are well into chronic infection, this is unlikely to fully account for the weaker HLA footprints on HIV in Mexico. Second, the cohort enrollment period was later for Mexico (2000 to 2014) than for Canada/United States (1996 to 2004) and HIV sequence diversity was higher, raising the possibility that the Mexican epidemic was older at the time of sampling than the Canada/United States one (61) and, thus, more preadapted to its host population (11, 62, 63). If so, this could reduce our overall ability to identify HAPs; however, we observed no strong evidence to support widespread preadaptation to all HLA alleles in Mexico (although evidence of HIV adaptation to certain common HLA alleles was indeed noted) (Fig. 8D). Furthermore, despite both epidemics being HIV subtype B, we cannot rule out the possibility that regional differences in viral backbone influence adaptation pathways. However, the phylogenetic intermixing of study HIV sequences and the observation of cohort consensus differences at only 7/934 (0.75%) HIV codons argues against this as a major confounder. It is also important to note that, when designating a particular HAP as shared versus unique to a given cohort, we are referring to HAPs identified at a q value of <0.2 in both versus only one cohort, respectively. HAPs unique to a given cohort may still be present in the other cohort above this significance threshold. Finally, we have not measured HLA-associated immune responses directly in this study; rather, we are using HLA footprint data to make inferences regarding the strength and reproducibility of HLA-restricted antiviral cellular immune responses in given host populations (7).

We propose some hypotheses as to why HLA-mediated pressures on HIV may be weaker in Mexico. First, it is possible that targeting of specific HLA-restricted CTL epitopes, and/or immunodominance hierarchies, are not as consistent in Mexico as in other populations. Host immunogenetic differences in genes encoding proteins that interact with HLA, in particular the T-cell receptor repertoire, also could explain differential recognition and/or escape within a given HLA-restricted CTL epitope across human populations (17). Indeed, our observation of substantial differential selection of HIV polymorphisms by HLA alleles present in both cohorts (Fig. 8 and 9) supports host factors beyond HLA in mediating these differences. Marked differences in HLA subtype distributions (e.g., the greater diversity of HLA-B*35 subtypes in Mexico than in Canada/United States) also may play a role, as closely related HLA alleles with similar or identical epitope binding motifs nevertheless may target epitopes at different frequencies with different functional avidities and elicit differential escape pathways (17, 64). The possibility of HLA locus-specific differences is also intriguing. Consistent with a dominant influence of HLA-B in mediating anti-HIV immune responses (65), >50% of HAPs identified in Mexico and Canada/United States were HLA-B restricted; however, whereas an average of 12 HAPs were identified per HLA-B allele in Canada/United States, only 2.8 HAPs were identified per HLA-B allele in Mexico. In contrast, the average number of HAPs per HLA-A and -C allele were only 2-fold lower in Mexico than in Canada/United States (e.g., 5 and 3.4 per HLA-A and HLA-C allele in Mexico and 11 and 7.8 in Canada/United States), raising the intriguing possibility that individual HLA-B alleles do not restrict as broad or potent an anti-HIV immune response in Mexico as elsewhere. Also intriguing was our observation of relatively strong positive relationships between HLA frequency and the number of HLA-restricted adapted HAPs in Canada/United States (Spearman's rho of 0.4098 and *P* = 0.003 for Gag and rho of 0.3358 and *P* = 0.0062 for PR-RT) but far less so in Mexico (*P* = 0.0954 and rho of 0.2137 for Gag and *P* = 0.0539 and rho of 0.2893 for PR-RT) (data not shown). Notable examples include B*35:01 (that restricts 3 adapted associations in Mexico and 12 in Canada/United States despite being present at comparable allele frequency) and A*02:01 (that restricts 2 adapted HIV associations in Mexico and 8 in Canada/United States despite being present at comparable frequency across cohorts). Converging selection pressures by different HLA alleles on the same HIV codon also may play a role. RT codon 135, for example, harbors diametrically opposed HAPs restricted by different HLA alleles (B*51:01-135T and B*15:03-135I); it is intriguing that the latter HAP is among the few that are significantly stronger in Mexico than in Canada/United States, which could conceivably influence the strength of the B*51:01-135T association in Mexico. Overall, our observations highlight the need for detailed assessments of HLA-restricted CTL responses, possibly supplemented with the characterization of T-cell receptor genetic and functional diversity in the Mexican mestizo population for select HLA-restricted HIV epitopes. Our observations also support extension of our analyses to other immunogenic HIV proteins, such as Nef, which exhibit high HAP densities (7, 14, 17, 54).

### Conclusions.

Comparative HLA footprint studies are relevant to HIV vaccine design because they illuminate the extent to which viral immunogenic regions, and their associated escape pathways, are universal versus population specific. Combined with information on sequence conservation, fitness costs, and escape mechanisms, HLA footprints can be used to identify immunogenic yet constrained viral regions, and their common sequence variants, for potential vaccine inclusion. In particular, HLA footprints can guide the discovery of novel epitopes and/or immunogenic regions (66, 67), which may be of particular importance in understudied populations with unique HLA distributions. HLA footprints may similarly prove useful in the context of therapeutic vaccinations for reservoir eradication (68), for example, by analyzing autologous HIV reservoir sequences to assess the burden of escape therein. Our study extends a growing body of evidence supporting both universal and population-specific HLA-associated footprints on HIV, even among neighboring epidemics where the same HIV subtype circulates. While the identification of shared immunogenic regions in Gag and Pol could support the notion of an HIV subtype B vaccine tailored to North American sequence diversity, the identification of novel HIV adaptation pathways restricted by typical mestizo HLA alleles and, more importantly, the unexpected observation of a significantly scarcer and weaker HLA footprint on HIV in Mexico raises intriguing questions regarding the strength and quality of HLA-restricted antiviral immunity in the Mexican mestizo population and what implications this might have for vaccine-induced immune responses. Thus, detailed characterizations of HLA-restricted CTL responses in this unique population are merited.

## MATERIALS AND METHODS

### Ethics statement.

This study was approved by the Ethics Committee of the National Institute of Respiratory Diseases (INER) in Mexico City (codes E02-05 and E10-10), the institution leading and coordinating the study, and was conducted according to the principles of the Declaration of Helsinki. All participants gave written informed consent before blood sample donation.

### Mexican cohort.

Antiretroviral-naive, chronically HIV-1 subtype B-infected Mexican individuals were enrolled from 2000 to 2014 as part of a national project to assess HIV molecular epidemiology, drug resistance surveillance, and HLA adaptation. Participants were enrolled by convenience sampling in HIV clinics and reference hospitals in Mexico City and the states of Baja California, Campeche, Chiapas, Chihuahua, Colima, Guerrero, Hidalgo, Jalisco, Michoacan, Morelos, Nuevo Leon, Oaxaca, Puebla, Queretaro, Quintana Roo, Sinaloa, Sonora, State of Mexico, Tabasco, Tlaxcala, Veracruz, and Yucatan. Each participant donated a single blood sample from which plasma and buffy coat/peripheral blood mononuclear cells were isolated and cryopreserved. All blood samples were processed at the Center for Research in Infectious Diseases (CIENI) of INER in Mexico City. HIV plasma viral load was determined with the m2000 system (Abbott, Abbott Park, IL, USA). CD4^+^ T-cell counts were determined by flow cytometry using the TruCount kit in a FACSCanto II instrument (BD Bioscience, San Jose, CA, USA).

### Reference Canada/United States cohort.

A reference population, comprising two published cohorts of antiretroviral treatment-naive, HIV-1 subtype B-infected individuals from Canada (the British Columbia Observational Medical Evaluation and Research [HOMER] cohort; *n* = 1,103) (16, 52) and the United States (AIDS Clinical Trials Group [ACTG] protocol 5142 participants who also provided human DNA under ACTG protocol 5128; *n* = 538) (53, 54), for whom HIV sequences linked to HLA class I types were available, was used as a comparison group. The Canada/United States cohort was chosen as a reference because the epidemics in these two countries and in Mexico are geographically linked, concentrated in persons with similar risk factors, and predominantly HIV subtype B. The Canada/United States cohorts, along with another from Australia, were previously used to identify HLA-associated polymorphisms in HIV subtype B (7); here, the Canada/United States cohorts were reanalyzed for HLA footprints specific to North America. As described previously (16, 52), the majority of HLA class I types were defined at subtype-level resolution; missing or intermediate-resolution data were imputed to subtype level using a machine-learning algorithm trained on HLA-A, -B, and -C subtypes from >13,000 individuals with known ethnicity (69). Extensive validations of method robustness to HLA imputations are provided in reference 7, as are instructions for access to paired HIV/HLA data from this cohort.

### HIV *gag* and *pol* amplification and sequencing in the Mexican cohort.

Viral RNA was isolated from cryopreserved plasma (1 ml) using the QIAamp viral RNA kit (Qiagen, Valencia, CA, USA). For *gag* amplification, primers 623Fi, AAATCTCTAGCAGTGGCGCCCGAACAG (HXB2 genomic nucleotide positions 623 to 649), and 2cRx (2826 to 2849) were used for the first-round reverse transcription-PCR (RT-PCR) (70) with a Super Script III OneStep RT-PCR kit (Invitrogen, Carlsbad, CA) and the following PCR conditions: 30 min at 55°C and 2 min at 94°C, followed by 35 cycles of 15 s at 94°C, 30 s at 55°C, and 2 min at 68°C, and finishing with 5 min at 68°C. Second-round products were obtained with primers G1, GCAGGACTCGGCTTGCTGAA (positions 691 to 710), and G10, TATCATCTGCTCCTGTATC (2343 to 2325), using Platinum *Taq* DNA polymerase (Invitrogen) and the following PCR conditions: 3 min at 94°C, followed by 35 cycles of 30 s at 94°C, 30 s at 56°C, and 2 min at 72°C, and finishing with 5 min at 72°C. All positive *gag* products confirmed by agarose gel electrophoresis were purified using a QIAquick PCR purification kit (Qiagen). Sequences were obtained with eight primers (G2F, GCGGCGACTGGTGAGTA [734 to 750]; GS1R, TTATCTAAAGCTTCCTTGGTGTCT [1074 to 1097]; GAS3F, CATCAATGAGGAAGCTGCAG [1401 to 1420]; GAS4R, GGTTCTCTCATCTGGCCTGG [1462 to 1481]; GAS5F, CTCTAAGAGCCGAGCAAGCT [1697 to 1716]; GAS6R, AAAATAGTCTTACAATCTGG [1771 to 1790]; HPR1977F, GTTAAGTGTTTCAATTGTGG [1957 to 1976]; and GA2274R, TCTTTATTGTGACGAGGGGTCG [2274 to 2295]) using BigDye v3.1 chemistry on a 3730xl genetic analyzer (Thermo Fisher, Waltham, MA, USA). Sequences were assembled and manually edited using Geneious v5.6.7 (Biomatters, Auckland, New Zealand) and then aligned using MEGA 7 software (71).

For *pol* (PR-RT) sequences, amplification of HIV protease (99 amino acids) and the first 335 amino acids of the RT was performed using a previously described in-house protocol (72). Sequences were obtained with a 3730xl genetic analyzer (Thermo Fisher) and assembled using the automated base-calling software RECall (73). Negative controls were included in all amplification runs and monthly phylogenetic controls were performed, including laboratory HIV strains, to detect possible contamination.

### HIV subtyping.

HIV subtypes were determined using the REGA HIV subtyping tool (3.0) (http://dbpartners.stanford.edu:8080/RegaSubtyping/stanford-hiv/typingtool/) and confirmed with the Recombination Identification Program (RIP; https://www.hiv.lanl.gov/content/sequence/RIP/RIP.html) (74). All non-subtype B sequences were removed prior to analysis.

### Phylogenetic analyses and cluster identification.

HIV *gag* and *pol* sequences were aligned to the HIV HXB2 reference strain using an in-house alignment algorithm based on HyPhy (75), and columns where HXB2 was gapped were stripped out. Shannon entropy of amino acid alignments was computed using the Los Alamos HIV sequence database (https://www.hiv.lanl.gov/content/sequence/ENTROPY/entropy.html) with 500 randomizations. Maximum likelihood phylogenies were inferred with FastTree (http://www.microbesonline.org/fasttree) using the generalized time-reversible (GTR) model (76, 77). Phylogenies were colored using Rainbow Tree (https://www.hiv.lanl.gov/content/sequence/RAINBOWTREE/rainbowtree.html) (78). Patristic distances were extracted from cohort-specific phylogenies using PATRISTIC (79). *gag* and *pol* sequence clusters, defined by within-cluster patristic distances of ≤1.5% and bootstrap support values of ≥90%, were identified using Cluster Picker (The University of Edinburgh, United Kingdom) (80). This genetic distance threshold has been used previously for inferring transmission clusters in chronic cohorts (81). HIV genetic compartmentalization between cohorts was assessed using the fixation index (F_ST_) score (82) implemented in HyPhy (75).

### HLA typing in the Mexican cohort.

Genomic DNA was extracted from a minimum of 6 million PBMC or 200 μl of buffy coat using a QIAmp DNA blood minikit (Qiagen). HLA class I HLA-A, -B, and -C typing was performed to subtype-level (4-digit) resolution using a modified in-house sequence-based method (83). Briefly, 1-kb fragments, including exons 2 and 3 of HLA-A, -B, and -C, were amplified using universal, locus-specific primers and the Roche Expand high-fidelity PCR system (Roche Applied Science, Laval, PQ, Canada). PCR products were cleaned up with ExoSAP-IT (Affimetrix, Cleveland, OH, USA) and sequenced on a 3730xl genetic analyzer using BigDye 3.1 chemistry (Thermo Fisher). HLA allele assignment was done using uTYPEv6 (Thermo Fisher) by comparison to the IMGT/HLA database (84, 85). Using this method, a total of 92 HLA-A, 91 HLA-B, and 39 HLA-C allele pairs within the Common and Well-Documented Catalogue (86) present polymorphism phase ambiguities at the resolution level of the first (i.e., allele-level) or second (i.e., subtype-level) HLA fields (see Table S1 in the supplemental material). These ambiguities were resolved by assigning the most frequent allele combination according to linkage disequilibrium data obtained from our Mexican mestizo population. Ambiguous HLA pairs due to polymorphic differences outside exons 2 and 3 were managed as G groups, including A*74:01:01G (A*74:01 in the analysis, encompassing A*74:01/A*74:02), C*18:01:01G (C*18:01 in the analysis, encompassing C*18:01/C*18:02), C*17:01:01G (C*17:01 in the analysis, encompassing C*17:01/C*17:02/C*17:03), and C*04:01:01G (C*04:01 in the analysis, encompassing C*04:01/C*04:09N), among others. All HLA haplotypes were confirmed using the HLA completion web tool (available at http://boson.research.microsoft.com/hla/) (69). Additionally, a total of 33 HLA-A or HLA-C types that failed amplification or sequencing were imputed using the same tool. HLA haplotypes with unresolved HLA-B loci were not imputed and were considered missing data (this included 8 individuals with both HLA-B alleles and 8 with one HLA-B allele missing). Raw HLA typing data are available via direct request to the authors.

Further validation of our HLA typing method in the context of a Mexican mestizo population was performed, analyzing HLA data from 323 individuals from Mexico City for whom HLA typing had been performed by amplifying exons 1 to 8 for HLA-A and HLA-C and exons 1 to 7 for HLA-B followed by next-generation sequencing (TruSight HLA kit; Illumina), thereby resolving gametic phase and achieving the highest possible typing resolution. In a blinded manner, we extracted the exon 2 and 3 consensus sequences (i.e., without gametic-phase resolution) from these patients and reinterpreted them as described above. Accuracy was 99.89% for HLA subtypes assigned by sequencing all exons with gametic-phase resolution versus exons 2 and 3 without gametic-phase resolution and those at four-digit resolution (only 1 out of 969 HLA loci was inaccurate). The results of this validation are shown in Table S2.

### HLA frequency comparison.

HLA allelic frequencies in Mexico and Canada/United States were compared using the Los Alamos HIV Database HLA comparison tool (https://www.hiv.lanl.gov/content/immunology/hla/hla_compare.html), which computes two-sided exact Fisher's test *P* values corrected for multiple comparisons using Storey's q value, which estimates the false discovery rate (87). Results with a *P* value of <0.05 and q value of <0.2 were deemed statistically significant.

### Identification and comparison of HLA-associated polymorphisms, including formal tests for differential escape between populations.

HLA-associated polymorphisms in HIV subtype B Gag and PR-RT were identified in the Mexico and Canada/United States cohorts separately using a published phylogenetically informed statistical model that corrects for potential host and viral genetic confounders, including HLA linkage disequilibrium, the HIV phylogeny, and HIV codon covariation (14). The model identifies two types of associations: adapted (viral amino acids overrepresented in individuals expressing the HLA allele, representing the inferred escape form) and nonadapted (viral amino acids underrepresented in individuals expressing the HLA allele, representing the inferred susceptible form). All associations with a q value of <0.2 were organized into immune escape maps. We also wished to compare the strengths of association of individual HAPs across the two cohorts. To do this, we took the union of all HAPs identified in Mexico and/or Canada/United States that were restricted by HLA class I alleles observed in a minimum of 10 individuals in both cohorts and applied a published phylogenetically corrected logistic regression to test whether their strengths of selection by the restricting HLA allele differed significantly between the cohorts (17, 64). Briefly, for each HAP of interest, the model computes a *P* value testing whether HLA-mediated selection is the same in Mexico and Canada/United States (null hypothesis) or whether selection differs between cohorts (alternative hypothesis) (17). As before, a q value of <0.2 was defined as the significance threshold for the differential escape analysis. Finally, to demonstrate that our HLA imputation/ambiguity resolution method does not significantly affect HAP recovery or association strength in the Mexican cohort, we repeat all analyses excluding all ambiguous and imputed HLA loci for this cohort and verified that all original observations still held (Table S3 and Fig. S1).

## Supplementary Material

Supplemental material
