# Supplementary material for: Weaker HLA Footprints on HIV in the Unique and Highly Genetically Admixed Host Population of Mexico
Source: J Virol. 2018 Jan 2;92(2):e01128-17. doi: 10.1128/JVI.01128-17 (PMC5752930; doi:10.1128/JVI.01128-17)
Supplement: Supplemental material [file supp_92_2_e01128-17__index.html]

Weaker HLA Footprints on HIV in the Unique and Highly Genetically Admixed Host Population of Mexico — Supplemental material 

# Weaker HLA Footprints on HIV in the Unique and Highly Genetically Admixed Host Population of Mexico

## Supplemental material

- Supplemental file 1 -

  Table S1 (Frequency of four-digit HLA-A, HLA-B, and HLA-C ambiguities in the Mexican cohort.)

  Table S2 (HLA-typing agreement using complete next-generation sequencing data versus that for exons 2 and 3 only.)

  Table S3 (HLA-associated polymorphisms in HIV-1 subtype B Gag and PR-RT in samples from Mexico, excluding HLA imputations/ambiguities.)

  Table S4 (Entropy differences per Gag codon between samples from Mexico and Canada/USA.)

  Table S5 (Entropy differences per PR-RT codon between samples from Mexico and Canada/USA.)

  Table S6 (HLA allelic frequency comparison between samples from Canada/USA and Mexico.)

  Table S7 (HLA-associated polymorphisms in HIV-1 subtype B Gag and PR-RT in samples from Mexico.)

  Table S8 (HLA-associated polymorphisms in HIV-1 subtype B Gag and PR-RT in samples from Canada/USA.)

  Table S9 (Strength of selection comparison for HLA-associated polymorphisms in Gag and PR-RT between samples from Mexico and Canada/USA.)

  XLSX, 422K
- Supplemental file 2 -

  Fig. S1 (Validation of results, excluding cases of HLA imputation/ambiguity.)

  PDF, 811K
