# Supplementary material for: Weaker HLA Footprints on HIV in the Unique and Highly Genetically Admixed Host Population of Mexico
Source: J Virol. 2018 Jan 2;92(2):e01128-17. doi: 10.1128/JVI.01128-17 (PMC5752930; doi:10.1128/JVI.01128-17)
Supplement: Supplemental material [file JVI.01128-17_zjv002183244s2.pdf]

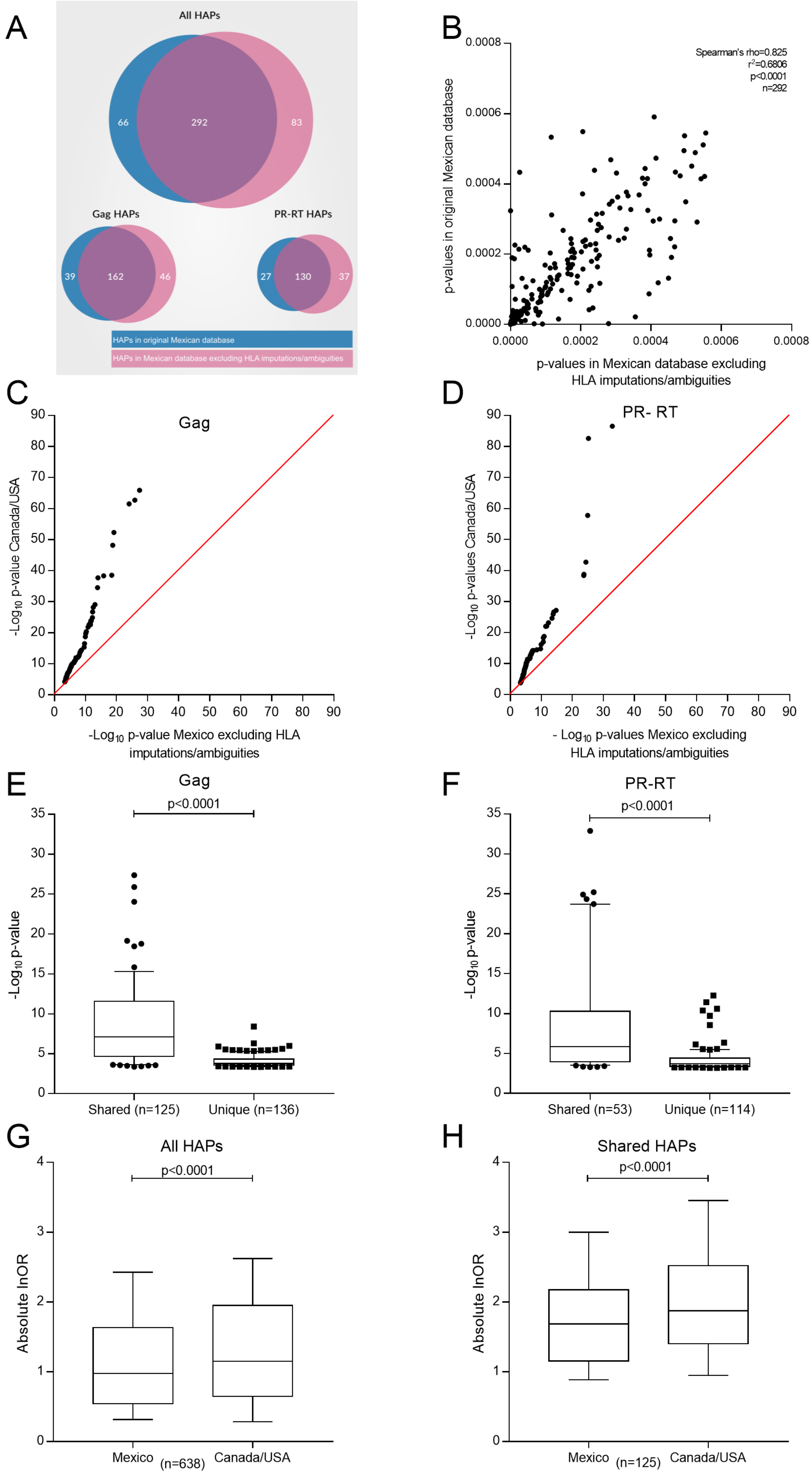

Figure S1. Validation of results excluding cases of HLA imputation/ambiguity. We identified all cases in the Mexican cohort where polymorphism phase ambiguities exist (n=255) or where HLA typing was imputed due to amplification/sequencing failure (n=33) and repeated all analyses in the original paper. The number and location of HLA-associated polymorphisms identified in the Mexican cohort are highly (>80%) consistent with those reported in the original analysis. Discrepancies in the range of ~20% are expected given our use of a q-value correction for multiple testing; at $q<0.2$ we expect ~20% of identified associations to be false positives (A). The p-values of HLA-associated polymorphisms identified in the original and revised analyses are highly concordant (Spearman's $r=0.825$, $p<0.0001$ (B). Results of the re-analysis fully corroborate our original observations of significantly fewer and weaker HLA-associated footprints in Mexico compared to Canada/USA (C-H). In fact, the association reported in panel F is even stronger than originally reported.
